# Daytime-restricted parenteral feeding is associated with earlier oral intake in children following stem cell transplant

**DOI:** 10.1172/JCI167275

**Published:** 2023-02-15

**Authors:** YunZu Michele Wang, Cynthia B. Taggart, John F. Huber, Stella M. Davies, David F. Smith, John B. Hogenesch, Christopher E. Dandoy

**Affiliations:** 1 Division of Bone Marrow Transplantation and Immune Deficiency, Cincinnati Children’s Hospital Medical Center, Cincinnati, Ohio, USA.; 2 University of Cincinnati College of Medicine, Cincinnati, Ohio, USA.; 3 Division of Pediatric Otolaryngology and; 4 Divisions of Human Genetics, Immunobiology, and Pulmonary Medicine, Cincinnati Children’s Hospital Medical Center, Cincinnati, Ohio, USA.

**Keywords:** Clinical Trials, Metabolism, Clinical practice, Stem cell transplantation

## To the editor:

In inpatients unable to sustain themselves enterally, standard i.v. total parenteral nutrition (TPN) is often administered over a 20- to 24-hour feeding period. However, feeding time is a dominant entraining cue for peripheral mammalian clocks and, importantly, a major driver of metabolism (1). It is well established that eating over long periods such as these contributes to metabolic dysregulation and poor sleep. Moreover, the first report of long-term TPN, given at home to adults with chronic bowel disease, was administered overnight for 12–18 hours (2). Time-restricted feeding is actively being studied in adult outpatients with obesity and those with healthy weight, with evaluation of weight loss or metabolic syndrome manifestations. The benefits of limiting the time of day and daily feeding duration in the inpatient setting in pediatric patients experiencing an acute but expected recoverable intestinal insult are unknown but likely important to study.

**Figure 1:**
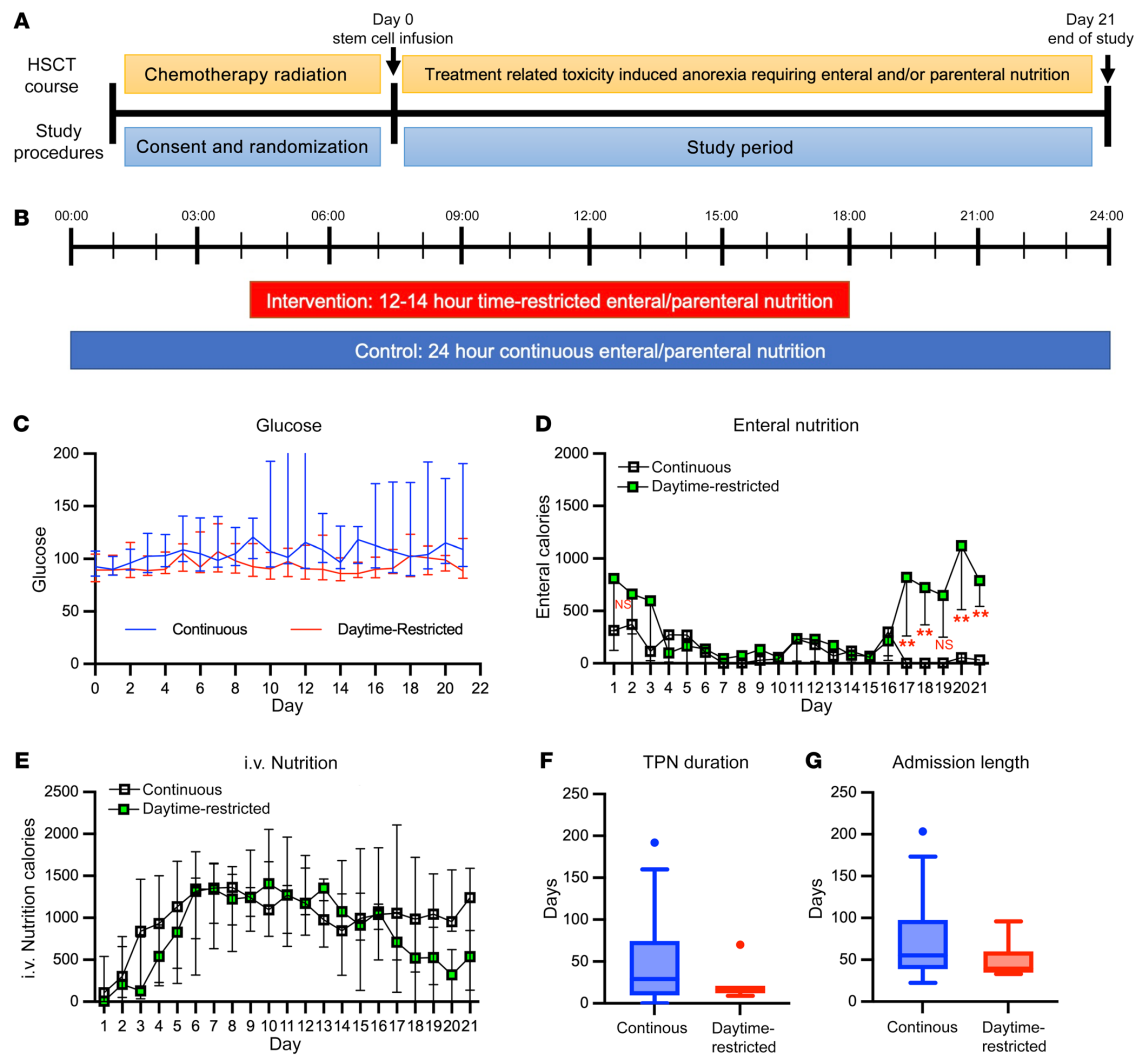
Daytime-restricted parenteral feeding is associated with earlier oral intake compared with continuous parenteral feeding in children after HSCT. (**A**) Study timeline. (**B**) Study schema. (**C**) Glucose level in daytime-restricted nutrition (red) and continuous nutrition (blue) groups. The *x* axis represents days after HSCT. (**D**) Enteral calories in daytime-restricted (green) and continuous (white) groups. **P < 0.01. (**E**) i.v. nutrition calories in daytime-restricted (green) and continuous (unfilled) groups. (**F**) Duration of total parenteral nutrition (TPN) administration starting at day 0 in continuous (blue) and daytime-restricted (red) groups (*P* = 0.618). (**G**) Inpatient admission length for HSCT (*P* = 0.315). Data represent median ± interquartile range in **C**–**G**. Statistical significance was determined by Mann-Whitney *U* test for all analyses in **D**–**G**.

We conducted a single-center, randomized, nonblinded, controlled pilot study from May 2020 to September 2022 to establish the safety and feasibility of daytime-restricted nutrition in hematopoietic stem cell transplant (HSCT) recipients and to evaluate the effect of nutritional supplementation timing on post-HSCT outcomes (ClinicalTrials.gov, NCT04549038). This study was approved by the Cincinnati Children’s Hospital Medical Center Institutional Review Board and performed in accordance with the principles of the Declaration of Helsinki. Patients or their legal guardians provided written informed consent. Study investigators enrolled participants during transplant conditioning, and study participation began on day 0 of HSCT (Figure 1A). Patients were excluded if they were under 1 year of age; received a reduced intensity preparative regimen; had a history of hypoglycemia, diabetes mellitus, or metabolic disease; or had another requirement for continuous nutrition. Enrolled participants were randomized 1:1 to either the control group, which received standard-of-care 20- to 24-hour continuous nutrition, or the intervention group, which received 12- to 14-hour (from 4 AM to 6 PM) enteral/parenteral nutrition for the first 21 days after HSCT (Figure 1B). Outcomes of interest included weight change, glucose levels, and time to tolerate oral feeding. Food logs and calorie counts were performed by registered dietitians and compared using *t* tests. Enrollment of 60 participants over two years was planned, but the study was stopped after limited enrollment during the COVID-19 pandemic and due to preliminary findings.

Eighteen participants were enrolled, including eight in the daytime-restricted nutrition arm (median age, 8.6 years [range, 6.7–15.7 years]; continuous arm median age, 11.1 years [range, 3.2–29.4 years]; Supplemental Table 1; supplemental material available online with this article; https://doi.org/10.1172/JCI167275DS1). All participants received 100% of their daily nutrition and electrolyte supplementation, and none experienced hypoglycemia. One participant in the intervention arm lost central access and required continuous i.v. fluids for seven days instead of TPN (Supplemental Figure 1). Weight changes after HSCT were not different between groups (data not shown). There were no differences in morning glucose values, but participants receiving continuous nutrition did have broader glucose ranges (Figure 1C). There were also no differences between oral and enteral caloric intake immediately after HSCT. Still, participants receiving daytime-restricted nutrition had significantly higher median enteral caloric intake on post-HSCT days +17–18 and 20–21 (day +20, *P* = 0.0036, Figure 1D). There were no changes in i.v. caloric intake (Figure 1E). In exploratory analyses, median TPN duration and inpatient admission were shorter in the time-restricted group (Supplemental Tables 2 and 3 and Figure 1, F and G, *P* = 0.618 and *P* = 0.315), and their variances were different (*P* = 0.0038 and *P* = 0.0119).

Daytime-restricted nutrition was safe and feasible in pediatric HSCT recipients and was associated with earlier oral/enteral intake. All study participants received their complete fluid, nutrition, and electrolytes within their assigned times, without the need for supplemental fluids or electrolyte repletion, with the exception of one participant, whose central line had to be removed (Supplemental Figure 1). There were no TPN-associated adverse events.

Hospital discharge after HSCT requires, at a minimum, adequate fluid, nutrition, and medication intake, so the similar patterns of TPN duration and hospital admission length were unsurprising. Considering the additional complications that may affect post-HSCT discharge readiness, particularly concerns for infection or graft-versus-host disease, a larger sample may have demonstrated a larger difference in admission length between the two groups. However, post-HSCT patients are often among the most acutely ill patients in a hospital outside the intensive care unit. The safety and feasibility of this intervention in our population corroborate those of earlier studies (3), particularly the findings of a study comparing cyclic to continuous TPN in adult HSCT recipients (4); we submit that they can be applied broadly to adult and pediatric inpatients. Furthermore, the 30%–50% of HSCT recipients who develop hyperglycemia immediately after transplant have increased risks of infection, intensive care hospitalization, and nonrelapse mortality (5). Early time-restricted feeding has been demonstrated to improve insulin sensitivity in prediabetic men, suggesting that our intervention has the potential to mitigate these risks in the post-HSCT population (6). Our study demonstrates earlier oral/enteral intake after HSCT in patients who received daytime-restricted nutrition, which may be due to the time of day limitation, as the adults in the prior study received TPN overnight, without differences in oral intake. These preliminary findings challenge the continuous TPN paradigm in hospitalized patients. We advocate that the potential metabolic, sleep, and healthcare utilization benefits of time-restricted feeding demand additional study.

## Supplementary Material

Supplemental data

## Version 1 02/15/2023

Electronic publication
