# Supplementary material for: Daytime-restricted parenteral feeding is associated with earlier oral intake in children following stem cell transplant
Source: J Clin Invest. 2023 Feb 15;133(4):e167275. doi: 10.1172/JCI167275 (PMC9927921; doi:10.1172/JCI167275)
Supplement: Supplemental data [file jci-133-167275-s074.pdf]

Supplemental material

Supplemental Table 1. Demographics and Bone Marrow Transplant Characteristics

Characteristic	**Continuous** (n=10)	**Time-Restricted** (n=8)
**Median Age (range)**	11.1 (3.2-29.4)	8.6 (6.7-15.7)
**Female**	4	6
**Underlying Diagnosis**		
Malignancy	6	5
Primary Immune Deficiency	1	0
Hemoglobinopathy	1	1
Storage Disorder	1	0
Bone Marrow Failure	1	2
**Allogeneic Transplant**	8	7
**Stem cell Source**		
Bone marrow	4	3
Peripheral blood stem cells	4	3
Umbilical cord blood	1	2
Bone marrow + Umbilical cord blood	1	0

Supplemental Table 2. Demographics and Bone Marrow Transplant Characteristics Stratified by Median TPN Duration of 19 days

**Characteristic**	**Continuous** (n=10)		**Time-Restricted** (n=8)	
	TPN<19 days (n=4)	TPN>19 days (n=6)	TPN<19 days (n=5)	TPN>19 days (n=3)
**Median Age (Range)**	17.4 (5.8-29.4)	8.7 (3.2-19.1)	7.9 (6.7-15.7)	9.5 (8.7-10.6)
**Female**	2 (50%)	2 (33%)	5 (100%)	1 (33%)
**Underlying Diagnosis**				
Malignancy	2 (50%)	4 (66%)	2 (40%)	3 (100%)
Primary Immune Deficiency	0	1 (17%)	0	0
Hemoglobinopathy	1 (25%)	0	1 (20%)	0
Storage Disorder	0	1 (17%)	0	0
Bone Marrow Failure	1 (25%)	0	2 (40%)	0
**Allogeneic Transplant**	2 (50%)	6 (100%)	4 (80%)	3 (100%)
**Stem cell Source**				
Bone marrow	0	4 (66%)	2 (40%)	1 (33%)
Peripheral blood stem cells	3 (75%)	1 (17%)	3 (60%)	0
Umbilical cord blood	0	1 (17%)	0	2 (67%)
Bone marrow + Umbilical cord blood	1 (25%)	0	0	0

Supplemental Table 3. Demographics and Bone Marrow Transplant Characteristics Stratified by Median Admission Length of 44.5 days

**Characteristic**	**Continuous** (n=10)		**Time-Restricted** (n=8)	
	Admission <44.5 days (n=4)	Admission >44.5 days (n=6)	Admission <44.5 days (n=5)	Admission >44.5 days (n=3)
**Median Age (range)**	9.1 (5.5-29.4)	14.2 (3.2-22.4)	7.9 (6.7-15.7)	8.7 (8.5-9.5)
**Female**	2 (50%)	2 (33%)	4 (80%)	2 (67%)
**Underlying Diagnosis**				
Malignancy	2 (50%)	4 (67%)	3 (60%)	2 (67%)
Primary Immune Deficiency	0	1 (17%)	0	0
Hemoglobinopathy	1 (25%)	0	0	1 (33%)
Storage Disorder	0	1 (17%)	0	0
Bone Marrow Failure	1 (25%)	0	2 (40%)	0
**Allogeneic Transplant**	3 (75%)	5 (83%)	4 (80%)	3 (100%)
**Stem cell Source**				
Bone marrow	1 (25%)	3 (50%)	2 (40%)	1 (33%)
Peripheral blood stem cells	2 (50%)	2 (33%)	3 (60%)	0
Umbilical cord blood	0	1 (17%)	0	2 (67%)
Bone marrow + Umbilical cord blood	1 (25%)	0	0	0

Supplemental Figure 1. CONSORT diagram. Figure created with BioRender.com

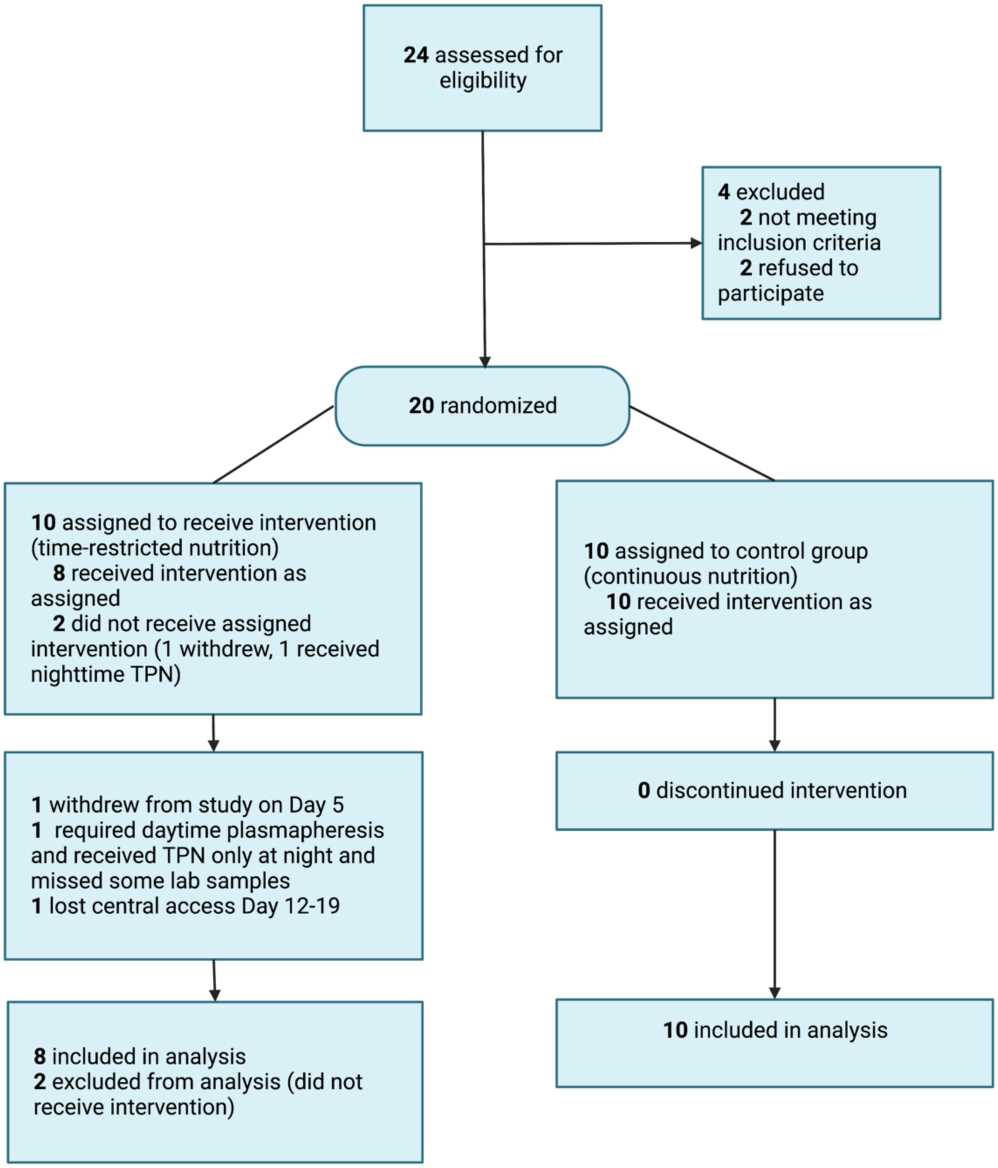

Statistics: The Mann-Whitney U test was used to compare medians across the two groups. F test was used to compare variances across the two groups for TPN duration and admission length. GraphPad Prism9 was used for all calculations.
